# Supplementary material for: Understanding and acceptance of the theory of evolution in high school students in Mexico
Source: PLoS One. 2023 Feb 8;18(2):e0278555. doi: 10.1371/journal.pone.0278555 (PMC9907822; doi:10.1371/journal.pone.0278555)
Supplement: S1 File — (DOCX) [file pone.0278555.s001.docx]

**Supporting information**

**S1 Table. I-SEA Questionnaire (Spanish version)**

**The answer format was a 5 level Likert scale with the following options: strongly disagree; disagree; neither agree nor disagree; agree; strongly agree.**

| 1. Pienso que las nuevas especies evolucionaron a partir de especies ancestrales. | 1. I think that new species evolved from ancestral species. |
| --- | --- |
| 2. Pienso que la evidencia fósil que los científicos usan para apoyar la teoría de la evolución es débil y no concluyente. | 2. I think that the fossil evidence that scientists use to support evolutionary theory is weak and inconclusive. |
| 3. Hay una gran cantidad de fósiles encontrados en todo el mundo que respaldan la idea de que los organismos evolucionan a nuevas especies con el tiempo. | 3. There are a large number of fossils found all around the world that support the idea that organisms evolve into new species over time. |
| 4. Pienso que todos los organismos complejos evolucionaron a partir de organismos unicelulares. | 4. I think all complex organisms evolved from single celled organisms. |
| 5. Pienso que las nuevas especies evolucionan a partir de una gran cantidad de pequeños cambios que ocurren durante períodos de tiempo relativamente largos. | 5. I think that new species evolve from a lot of small changes occuring over relatively long periods of time. |
| 6. Hay poca o ninguna evidencia observable para apoyar la teoría que describe cómo una especie de organismo evoluciona a partir de una forma ancestral diferente | 6. There is little or no observable evidence to support the theory that describes how one species of organism evolves from a different ancestral form . |
| 7. Las formas y diversidad de organismos han cambiado drásticamente con el tiempo | 7. The forms and diversity of organisms have changed dramatically over time. |
| 8. Creo que todos los organismos están relacionados (o comparten un ancestro común). | 8. I think that all organisms are related (or share a common ancestor). |
| 9. Pienso que los organismos, tal como existen ahora, están perfectamente adaptados a sus entornos naturales y, por lo tanto, no seguirán cambiando | 9. I think that organisms, as they exist now, are perfectly adapted to their natural environments and so will not continue to change. |
| 10. Todos los grupos de organismos seguirán cambiando | 10. All groups of organisms will continue to change. |
| 11. Hay una gran cantidad de ejemplos de organismos que han experimentado cambios evolutivos dentro de la especie (es decir, resistencia a los antibióticos en las bacterias, producción de nuevas cepas del virus de la gripe). | 11. There are a large number of examples of organisms that have undergone evolutionary changes within the species (i.e., antibiotic resistance in bacteria, production of new strains of the flu virus). |
| 12. Las especies fueron creadas para adaptarse perfectamente a su entorno, por lo tanto, no cambian. | 12. Species were created to be perfectly suited to their environment, so do not change. |
| 13. Yo no acepto la idea de que una especie de organismo desarrollará nuevos rasgos con el tiempo. | 13. I don't accept the idea that a species of organism will evolve new traits over time. |
| 14. Pienso que hay una gran cantidad de evidencia observable para apoyar la teoría que describe cómo pueden ocurrir variaciones dentro de una especie. | 14. I think there is an abundance of observable evidence to support the theory describing how variations within a species can happen. |
| 15. Las especies existen hoy exactamente en el mismo aspecto y forma en que siempre han existido. | 15. Species exist today in exactly the same shape and form in which they always have. |
| 16. Existe evidencia abrumadora que apoya la teoría de la evolución para explicar cómo ocurren las variaciones en una especie a lo largo del tiempo. | 16. There is overwhelming evidence supporting the theory of evolution to explain how variations in a species happen over time. |
| 17. Existe evidencia confiable que apoya la teoría que describe cómo los humanos se derivaron de los primates ancestrales. | 17. There is reliable evidence to support the theory that describes how humans were derived from ancestral primates. |
| 18. Aunque los humanos pueden adaptarse, los humanos no han evolucionado / no evolucionan. | 18. Although humans may adapt, humans have not/do not evolve. |
| 19. Pienso que las estructuras físicas de los humanos son demasiado complejas para haber evolucionado. | 19. I think the physical structures of humans are too complex to have evolved. |
| 20. Pienso que los humanos y los simios comparten un ancestro antiguo. | 20. I think that humans and apes share an ancient ancestor. |
| 21. Pienso que los humanos evolucionan | 21. I think humans evolve. |
| 22. Los humanos no evolucionan; solo pueden cambiar su comportamiento. | 22. Humans do not evolve; they can only change their behavior. |
| 23. Las muchas características que los humanos comparten con otros primates (es decir, chimpancés, gorilas) se pueden explicar mejor si compartimos un ancestro común. | 23. The many characteristics that humans share with other primates (i.e., chimpanzees, gorillas) can best be explained by our sharing a common ancestor. |
| 24. Las variaciones físicas en los seres humanos (es decir, color de ojos, color de piel) se derivaron de los mismos procesos que producen variaciones en otros grupos de organismos. | 24. Physical variations in humans (i.e., eye color, skin color) were derived from the same processes that produce variations in other groups of organisms. |

**S2 Table. KEE Questionnaire (Spanish version)**

| 1. ¿Cuál de los siguientes enunciados respalda la teoría de la evolución?  a. Bioquímica comparativa, donde se pueden cuantificar las similitudes y diferencias de ADN entre especies.  b. Embriología comparada, donde a menudo se puede rastrear la historia evolutiva de estructuras similares.  c. Selección artificial (también conocida como cría selectiva), un análogo de la selección natural.  d. Estructuras vestigiales que no tienen ningún propósito aparente.  e. Todas las anteriores proporcionan evidencia para apoyar la teoría de la evolución. | 1.Which of the following support the theory of evolution?  a. Artificial selection (also known as selective breeding), an analogue of natural selection.  b. Comparative biochemistry, where similarities and differences of DNA among species can be quantified.  c. Vestigial structures that serve no apparent purpose.  d. Comparative embryology, where the evolutionary history of similar structures can often be traced.  e. All of the above provide evidence to support the theory of evolution. |
| --- | --- |
| 2. La resistencia a una amplia variedad de insecticidas ha evolucionado recientemente en muchas especies de insectos. ¿Por qué?  a. Los seres humanos están alterando el medio ambiente de estos organismos y los organismos están evolucionando por selección natural  b. Los seres humanos tienen mejores prácticas de salud, por lo que estos organismos están tratando de mantenerse al día.  c. Las mutaciones van en aumento.  d. No están evolucionando nuevas especies, solo cepas o variedades resistentes. Esto no es evolución por selección natural.  e. Los insectos son más inteligentes que los humanos. | 2. Resistance to a wide variety of insecticides has recently evolved in many species of insects. Why?  a. Mutations are on the rise.  b. Humans are altering the environments of these organisms, and the organisms are evolving by natural selection.  c. No new species are evolving, just resistant strains or varieties. This is not evolution by natural selection.  d. Humans have better health practices, so these organisms are trying to keep up.  e. Insects are smarter than humans. |
| 3. ¿Cuál de las siguientes situaciones es la más exitosa en un sentido evolutivo?  a. Un león que logra capturar presas, pero no tiene cachorros  b. Un león que tiene muchos cachorros, ocho de los cuales viven hasta la edad adulta.  c. Un león que supera una enfermedad y vive para tener tres cachorros.  d. Un león que cuida a sus cachorros, dos de los cuales viven hasta la edad adulta.  e. Un león que tiene un harén de muchas leonas y un cachorro | 3. Which of the following is the most fit in an evolutionary sense?  a. A lion who is successful at capturing prey but has no cubs.  b. A lion who has many cubs, eight of which live to adulthood.  c. A lion who overcomes a disease and lives to have three cubs.  d. A lion who cares for his cubs, two of whom live to adulthood.  e. A lion who has a harem of many lionesses and one cub. |
| 4. ¿Cómo podría un biólogo explicar por qué una especie de ave ha desarrollado un tamaño de pico más grande?  a. El gran tamaño del pico ocurrió como resultado de la mutación en cada miembro de la población.  b. Los antepasados de esta especie de ave encontraron un árbol con semillas de tamaño más grande que el promedio. Necesitaban desarrollar picos más grandes para comer las semillas más grandes y, con el tiempo, se adaptaron para satisfacer esta necesidad.  c. Algunos miembros de la población ancestral tenían picos más grandes que otros. Si un pico de mayor tamaño fuera ventajoso, sería más probable que sobrevivieran y se reprodujeran. Como tal, las aves de pico grande aumentaron en frecuencia en relación con las aves de pico pequeño  d. Los antepasados de esta especie de ave encontraron un árbol con semillas de tamaño mayor al promedio. Descubrieron que, al estirar sus picos, los picos se alargaban y este aumento se transmitía a sus crías. Con el tiempo, los picos de las aves se hicieron más grandes.  e. Ninguna de las anteriores. | 4. How might a biologist explain why a species of birds has evolved a larger beak size?  a. Large beak size occurred as a result of mutation in each member of the population.  b. The ancestors of this bird species encountered a tree with larger than average sized seeds. They needed to develop larger beaks to eat the larger seeds, and over time, they adapted to meet this need.  c. Some members of the ancestral population had larger beaks than others. If larger beak size was advantageous, they would be more likely to survive and reproduce. As such, large-beaked birds increased in frequency relative to small-beaked birds.  d. The ancestors of this bird species encountered a tree with larger than average sized seeds. They discovered that by stretching their beaks, the beaks would get longer, and this increase was passed on to their offspring. Over time, the bird beaks became larger.  e. None of the above. |
| 5. ¿Cuál de las siguientes afirmaciones sobre la selección natural es verdadera?  a. La selección natural hace que surjan variaciones dentro de una población.  b. La selección natural conduce a una mayor probabilidad de supervivencia de ciertos individuos según la variación. La variación proviene de fuera de la población  c. Todos los individuos de una población tienen las mismas posibilidades de supervivencia y reproducción. La supervivencia se basa en elección  d. La selección natural da como resultado que aquellos individuos dentro de una población que están mejor adaptados sobrevivan y produzcan más descendencia.  e. La selección natural conduce a la extinción. | 5. Which of the following statements about natural selection is true?  a. Natural selection causes variation to arise within a population.  b. Natural selection leads to increased likelihood of survival for certain individuals based on variation. The variation comes from outside the population.  c. All individuals within a population have an equal chance of survival and reproduction. Survival is based on choice.  d. Natural selection results in those individuals within a population who are best adapted surviving and producing more offspring.  e. Natural selection leads to extinction. |
| 6. Todos los organismos comparten el mismo código genético. Esta similitud es evidencia de que:  a. La evolución está ocurriendo ahora.  b. Se ha producido una evolución convergente.  c. La evolución ocurre gradualmente.  d. Todos los organismos descienden de un ancestro común.  e. La vida empezó hace millones de años. | 6. All organisms share the same genetic code. This commonality is evidence that  a. Evolution is occurring now.  b. Convergent evolution has occurred.  c. Evolution occurs gradually.  d. All organisms are descended from a common ancestor.  e. Life began millions of years ago. |
| 7. ¿Cuál de las siguientes afirmaciones sobre la evolución por selección natural es falsa?  a. La selección natural actúa sobre los individuos.  b. La selección natural es un proceso aleatorio  c. Ventajas selectivas muy pequeñas pueden producir grandes efectos a lo largo del tiempo.  d. La selección natural puede resultar en la eliminación de ciertos alelos del acervo genético de una población.  e. Las mutaciones son importantes como la fuente última de variabilidad genética sobre la que puede actuar la selección natural. | 7. Which of the following statements regarding evolution by natural selection is false?  a. Natural selection acts on individuals.  b. Natural selection is a random process.  c. Very small selective advantages can produce large effects through time.  d. Natural selection can result in the elimination of certain alleles from a population’s gene pool.  e. Mutations are important as the ultimate source of genetic variability upon which natural selection can act |
| 8. Un cambio en la composición genética de una población de organismos a través del tiempo es:  a. Radiación adaptativa  b. Evolución biológica  c. Evolución Lamarckiana  d. Selección natural  e. Recombinación genética | 8. A change in the genetic makeup of a population of organisms through time is  a. Adaptive radiation.  b. Biological evolution  c. Lamarckian evolution.  d. Natural selection  e. Genetic recombination. |
| 9. ¿Cuál es la principal fuente de variación nueva en las poblaciones naturales?  a. Recombinación.  b. Mutación.  c. Hibridación  d. Flujo génico  e. Selección natural | 9. Which of the following is the ultimate source of new variation in natural populations?  a. Recombination.  b. Mutation  c. Hybridization.  d. Gene flow.  e. Natural selection |
| 10. ¿Cuál de las siguientes opciones describe mejor la relación entre evolución y selección natural?  a. La selección natural es un mecanismo que puede resultar en el proceso de evolución.  b. La selección natural produce cambios a pequeña escala en las poblaciones, mientras que la evolución produce cambios a gran escala.  c. La selección natural es un proceso aleatorio, mientras que la evolución avanza hacia un objetivo específico.  d. La selección natural es la supervivencia diferencial de poblaciones o grupos, lo que resulta en la evolución de organismos individuales.  e. Son términos equivalentes que describen el mismo proceso. | 10. Which of the following best describes the relationship between evolution and natural selection?  a. Natural selection is one mechanism that can result in the process of evolution.  b. Natural selection produces small-scale changes in populations, whereas evolution produces large-scale ones.  c. Natural selection is a random process whereas evolution proceeds toward a specific goal  d. Natural selection is differential survival of populations or groups, resulting in the evolution of individual organisms.  e. They are equivalent terms describing the same process. |

**S3 Table. I-SEA results per question.** Percentage of people who accept or reject the evolution by item

| **Ítems** | **High** | **Moderate** | **Low** | **Evolution sublevel** |
| --- | --- | --- | --- | --- |
| Question 1: | 75.7 | 14 | 10.3 | Macroevolution |
| Question 2*: | 67.6 | 17 | 15.4 |  |
| Question 3: | 84.9 | 10.2 | 4.9 |  |
| Question 4: | 70.3 | 21.1 | 8.6 |  |
| Question 5: | 83.2 | 12.7 | 4.1 |  |
| Question 6: * | 48.7 | 36.2 | 15.1 |  |
| Question 7: | 79.5 | 17 | 3.5 |  |
| Question 8: | 56.8 | 28.9 | 14.3 |  |
| Question 9*: | 56.2 | 21.1 | 22.7 | Microevolution |
| Question 10: | 81.9 | 13.5 | 4.6 |  |
| Question 11 | 81.3 | 14.9 | 3.8 |  |
| Question 12* | 59.2 | 19.2 | 21.6 |  |
| Question 13* | 75.1 | 13 | 11.9 |  |
| Question 14 | 72.2 | 22.4 | 5.4 |  |
| Question 15* | 31.9 | 12.7 | 55.4 |  |
| Question 16 | 71.1 | 23.5 | 5.4 |  |
| Question 17 | 66.8 | 28.1 | 5.1 | Human Evolution |
| Question 18* | 65.4 | 19.7 | 14.9 |  |
| Question 19 | 60 | 22.2 | 17.8 |  |
| Question 20 | 67 | 25.4 | 7.6 |  |
| Question 21 | 81.3 | 14.1 | 4.6 |  |
| Question 22* | 66.5 | 21.3 | 12.2 |  |
| Question 23 | 67 | 25.7 | 7.3 |  |
| Question 24 | 64.9 | 28.1 | 7 |  |

**Fig S1. I-SEA results per question.** Percentage of people who accept or reject the evolution by item. Graphical representation of the information contained in Table S3.
