# Supplementary material for: Understanding and acceptance of the theory of evolution in high school students in Mexico
Source: PLoS One. 2023 Feb 8;18(2):e0278555. doi: 10.1371/journal.pone.0278555 (PMC9907822; doi:10.1371/journal.pone.0278555)
Supplement: Attachment — Submitted filename: Response to reviewer.docx [file pone.0278555.s002.docx]

September 8^th^, 2022.

Dr. Thomas Tischer

Staff Editor, PLOS ONE

Thank you for your correspondence of August 31^st^, regarding our paper titled “*Understanding and acceptance of the theory of evolution in high school students in Mexico*” (ID: PONE-D-22-19895).

We thank the Reviewer for carefully reading our manuscript and his suggestions for improving the paper. In summary, the Reviewer recognized the significance, relevance, comprehensiveness, and scientific rigor of our work, and provided us with minor comments that gave us the opportunity to improve some aspects of our paper.

We addressed all the Reviewer’s remarks in our point-by-point reply and modified our manuscript according to the Reviewer’s constructive comments. All changes are highlighted in the revised manuscript.

We believe that, as a result of this review, the quality of the presentation has improved to reach the publication standards of PLOS ONE.

Finally, given the minor character of the modifications requested by the Reviewer, we would like to respectfully ask for a speedy processing of our revised paper. The reason being that the institutional funding that we secured to cover the article processing charge needs to be spent before the end of the fiscal year, with an estimated deadline by the end of this month. We thank you in advance for your comprehension and attention to this matter.

We look forward to receiving your decision.

Respectfully,

Dr. Gonzalo Peñaloza Jiménez

**Reviewer #1:** The manuscript was focused on the understanding and acceptance theory of evolution. Authors used validated research tools. Authors used quantitative approach toward obtaining and analyzing of data. The statistical methods were used adequate. The text is divided into chapters typical for empirical study. The text is written in understandable form and also it is written on high level. Below are comments which have got minor character. The comments are presented below.

We thank the Reviewer for the reading our manuscript and for providing us with pertinent recommendations that allowed us improving the presentation of our work. We have addressed all the comments; all changes can be found highlighted in the revised manuscript.

1. My comments are toward methodological part of the manuscript:

- subchapter “Data analysis” includes kinds of information, which are only informative, for example kinds of information about values of Pearson product moment, what is low and what is high correlation. So please eliminate these kinds of information.

We thank the Reviewer for this observation. We have modified this part of the manuscript and left only the essential information.

- nowadays, the better statistical methods for the determination of reliability is Cronbach’s alpha coefficient. Even for multiple-choice questions of the test.

We thank the Reviewer for this remark. We performed the calculation of the Cronbach’s alpha coefficient for the KEE questionnaire and included the results in the revised manuscript. This reliability value is in good agreement with that obtained using the Kuder-Richardson formalism.

- please add the normality evaluation. It confirms the using of selected methods.

We are particularly thankful to the Reviewer for this comment. Indeed, the verification that the data follows a normal distribution was missing; we apologize for this omission. This information is now included in the revised manuscript.

Specifically, in the revised manuscript we report the results of numerical fittings to standard Gaussian distributions of:

i) the scores of I-SEA and KEE for the entire population (Fig. 1 of the revised manuscript).

ii) the scores of I-SEA and KEE after categorizing them based on the students’ religious identity (Fig. 2 of the revised manuscript).

iii) the scores of KEE after categorizing them based on the education level of their mother and father (Fig. 3 of the revised manuscript).

These sets of data are the ones that were used in the statistical tests (t-test and ANOVA), and the distributions show a good normality in general. The only distribution that moves away from an ideal Gaussian distribution is that of the KEE scores of nonreligious students (Fig. 2(b) in the revised manuscript). In this regard, we note that the variance-based statistical tests that we used are robust against deviations from a normal distribution; as long as the variance of the groups are similar, the more important requirement is the independence of the groups [R1-R5], which is satisfied in our case. Based on this argument, our selection of statistical tests is valid. We have clarified this in the revised manuscript and included pertinent references.

[R1] Pearson (1931). The analysis of variance in cases of non-normal variation. Biometrika, 114-133.

[R2] Box (1953). Non-normality and tests on variances. Biometrika, 40(3/4), 318-335.

[R3] Glass, et al., (1972). Consequences of failure to meet assumptions underlying the fixed effects analyses of variance and covariance. Review of educational research, 42(3), 237-288.

[R4] Harwell, et al., (1992). Summarizing Monte Carlo results in methodological research: The one-and two-factor fixed effects ANOVA cases. Journal of educational statistics, 17(4), 315-339.

[R5] Lix, et al., (1996). Consequences of assumption violations revisited: A quantitative review of alternatives to the one-way analysis of variance F test. Review of educational research, 66(4), 579-619.

2. The results part of the manuscript includes some kinds of information, which regarding to methodology pat like information about reliability.

We thank the Reviewer for this observation. We have placed that information in the correct section in the revised manuscript.

3. Please revise References according to guidelines for authors.

We thank the Reviewer for this observation. In the revised manuscript, we have amended the format of the references according to the journal’s requirements.
