# Supplementary material for: Understanding and acceptance of the theory of evolution in high school students in Mexico
Source: PLoS One. 2023 Feb 8;18(2):e0278555. doi: 10.1371/journal.pone.0278555 (PMC9907822; doi:10.1371/journal.pone.0278555)
Supplement: Attachment — Submitted filename: reply to reviewer 2nd round+G-RG.docx [file pone.0278555.s003.docx]

Reviewer #2:

The manuscript seems well organized and logically structured traditionally according to IMRAD form. It is written clearly enough to be accessible to non-specialists. Authors have a broad outlook about the field they are interested in, which is declared by 70 references predominantly from the last two decades. The main claims of the paper are relevant and contribute to the dissemination of knowledge about students’ acceptance and understanding of evolutionary theory.

We thank the Reviewer for reading our manuscript and providing us with comments that allowed us improving the presentation of our work. We have addressed all the comments; all changes can be found highlighted in the revised manuscript.

The approach to solving the problem is not original. A similar view on the field of study has e.g. Kuschmierz et al. (2020) searching the students´ acceptance and understanding of evolutionary theory across Europe. This study can bring new information about Mexican students’ attitudes and knowledge in this subject matter. In the research, there were used two instruments: the I-SEA questionnaire and the KEE test, but in 2.3 part the MATE was mentioned too, probably, because the scale in the I-SEA questionnaire was adapted to MATE categories according to Kuschmierz et al. (2020, p.6), who remarked that “*... the I-SEA, we did not find any suggestions for categories in the original publication, which is why we suggested categories based on the MATE*”. What was the reason to choose these two instruments, if there are so many others specialized in the acceptance and understanding of the evolutionary theory/ the theory of evolution? The authors did not give adequate reasons for it.

As explained in our manuscript, one of our goals was to measure the acceptance and knowledge of the TE as independently as possible. The combination of questionnaires used serve this purpose and allows for different types of analysis. For instance, one can identify factors that influence the acceptance and knowledge separately; in our case, we found that religious identity was the only factor, among those considered, that affected the acceptance while the knowledge was affected by both religious identity and the parents’ education. Also, one can reliably stablish the existence of a relation between acceptance and knowledge. In this regard, some questions in MATE assess aspects of acceptance and understanding simultaneously, which can cause inconsistencies when studying the relation between them e.g., generation of stronger correlations. Finally, I-SEA evaluates different sublevels of the TE (microevolution, macroevolution, and human evolution). This was important for us because 1) for the Mexican context, there is not much information about the acceptance of the TE e.g., what aspects are more accepted/rejected, and 2) it allows using the information collected for pedagogic purposes e.g., to implement specially designed classroom interventions. We have clarified this in the discussion of the revised manuscript.

Another problem could be observed in using the second instrument (the KEE test). If there was used KEE test proposed by Nadelson and Southerland (2012) as it is cited in the manuscript, then the items/statements differ in the original instrument and instrument used by the authors. They were not verbatim taken over. It seems like pettiness, but...

for example (part 4.1 in manuscript versus Nadelson and Southerland (2012, p. 1655-1656)):

- There is little or no observable evidence to support the theory that describes how a species of organism evolves from a different ancestral form.
- I think that organisms, as they exist now, are perfectly adapted to their natural environments and, therefore, will not continue to change.
- The species were created to adapt perfectly to their environment, therefore they do not change.
- I do not accept the idea that a species of organism will develop new traits over time.
- Species exist today in exactly the same appearance and form in which have always existed.
- Although humans can adapt, humans have not/do not evolve.

in original

- There is little or no observable evidence to support the theory that describes how one species of organism evolves from another ancestral form.
- I think that organisms, as they exist now, are perfectly adapted to their natural environments and so will not continue to change.
- Species were created to be perfectly suited to their environment, so they do not change.
- I don´t accept the idea that a species of organism will evolve new traits over time.
- Organisms exist today in exactly the same form in which they always have.
- Although humans may adapt, humans have not/do not evolve.

We thank the Reviewer for this comment. The questionnaires in English we used were the original versions reported by their respective authors (KEE: Moore et al., 2009; I-SEA: Nadelson and Southerland, 2012). The comparison made above by the Reviewer is based on questions that were neither written nor reported by us (we suspect that the Reviewer translated our Spanish questionnaire back to English and assumed that we used that new English version). The questionnaires in Spanish were generated from three independent translations made by us, considering a natural Mexican phrasing while preserving the original sense of the questions. We have clarified this in the revised manuscript.

The other question is, whether the Spanish translation was correct. It is not elaborated, on how many of the participants solved the I-SEA questionnaire and the KEE test in English and how many in Spanish language. Wasn´t any difference in some parameters of them? Then it is questionable whether the data and analysis support the claims.

We thank the Reviewer for this comment and apologize for our omission. The questionnaires in English and Spanish were answered by 244 and 160 students, respectively. T-tests were performed on the results of the two groups (English and Spanish), and no significant differences were found (α=0.05, p=0.65 for I-SEA; α=0.05, p=0.14 for KEE), indicating that the performance of the students was similar irrespectively of the language of the questionnaire. We have included this information in the revised manuscript.

The following statement is in part 2.4. (Data analysis) not fortunately chosen: “*Extensive psychometric analysis was done to validate the use of these Instruments. For both instruments (the I-SEA and the KEE) Cronbachs’ alpha was used*”. Was the Cronbachs’ alpha used for the validity of the instrument?

Yes, both instruments were validated using Cronbach’s α (alpha) as a metric of reliability and internal consistency. For KEE, additionally, the Kuder-Richardson test was performed as it is recommended for multiple-choice tests where each item is scored binarily (correct/incorrect). We have clarified this in the revised manuscript.

Was the testing of data normality meaningful? What kind of data can be obtained processing the I-SEA questionnaire and the KEE test? What kind of variables are tested there? Many researchers argued, that the Likert scale can not generate normally distributed data. What about a multi-item Likert scale? Was appropriate to calculate a Pearson correlation coefficient?

We thank the Reviewer for this comment. We would like to note that the justification of our statistical analysis was amply discussed in the first review. In fact, one major modification in our revised manuscript, was the addition of all details about the normality tests, at the request of the first Reviewer.

Testing the normality of the data constitutes the validation of our choice of analysis. As the Reviewer correctly points out, there can be situations where the data are not normally distributed; in such cases, the tests carried out would be compromised (especially ANOVA and T-test), and a different analysis would be required.

I consider the formal mistake, that several titles are the same in different parts of it (e.g. Acceptance of evolution 1.1., 3.1., 4.2.; Understanding evolution 1.2, 3.2, 4.1; Relationship between acceptance and understanding 1.3, 3.3., 4.3.). In the table 3 the last column is signed “*T de student*”, in the Conclusions part there is written “*...there may be differences between macroevolution and macroevolution*” (?)

We thank the Reviewer for this comment. We have corrected this in the revised manuscript.

Barnes et al (2019) (who were not cited in the manuscript) advised of the fact that “*... the choice of the instrument used to measure evolution acceptance can influence the results and conclusions of a study*.” and “*Different instruments will measure different aspects of evolution acceptance and may then influence the conclusions that researchers will draw*.” Also, the I-SEA questionnaire and examining its sub-scales are cited in this context. This can bring a problem in the research procedure described in this manuscript, which could not be adequately and credibly reproduced.

Thank you for referring us to the work of Barnes et al (2019); we found that reference very useful to improve out manuscript. As pointed out by Barnes et al, by using a certain instrument, one implicitly assumes the definition of the constructs used for its development. I-SEA is not the exception as it is based on a particular definition of *acceptance*. In the revised manuscript, we have explicitly stated such definition and advised the reader to consider the differences in the definition of *acceptance* when comparing the results obtained by using different instruments. In this sense, it is important to mention that Barnes et al. affirm that no test is more effective than others; they simply advise researchers to be aware of the constructs assumed by each test and its limitations, as we have done in our revised manuscript. Also, Barnes et al. (2019) state that only a few research studies have been carried out using I-SEA. In this sense, we consider that our paper contributes to exploring its use in contexts and populations where it has not been utilized. Moreover, Barnes et al. (2019) pointed out that a positive relation between understanding and acceptance is often encountered when MATE is used. This might be due to some questions in MATE assess aspects of acceptance and understanding simultaneously, which can create an artificial correlation because the instruments overlap and evaluate the same aspect. In our case, we found a positive relationship between understanding and acceptance after measuring them independently, which can be considered a reliable result.

We have clarified the above in the revised manuscript, including our criteria to select the tests and an explanation why I-SEA was adequate for us (it allows disclosing the level of acceptance/rejection in the Mexican context and designing ad hoc classroom interventions; see comment above).

Conclusion: The manuscript is unsuitable for publication in its present form, but the study itself disposes of a potential that the authors could re-evaluate and choose relevant data for analysis and then resubmit a revised version.

Once again, we thank the Reviewer for reading our manuscript and providing us with feedback that allowed us improving the presentation of our work.
